# Searching iron sensors in plants by exploring the link among 2′-OG-dependent dioxygenases, the iron deficiency response and metabolic adjustments occurring under iron deficiency

**DOI:** 10.3389/fpls.2013.00169

**Published:** 2013-05-31

**Authors:** Gianpiero Vigani, Piero Morandini, Irene Murgia

**Affiliations:** ^1^Dipartimento di Scienze Agrarie ed Ambientali-Produzioni, Territorio, Agroenergia, Università degli Studi di MilanoMilano, Italy; ^2^Dipartimento di Bioscienze, Università degli Studi di MilanoMilano, Italy

**Keywords:** *Arabidopsis thaliana*, iron sensor, HIF (hypoxia-inducible factor), 2′-OG-dependent dioxygenase, prolyl 4-hydroxylase

## Abstract

Knowledge accumulated on the regulation of iron (Fe) homeostasis, its intracellular trafficking and transport across various cellular compartments and organs in plants; storage proteins, transporters and transcription factors involved in Fe metabolism have been analyzed in detail in recent years. However, the key sensor(s) of cellular plant “Fe status” triggering the long-distance shoot–root signaling and leading to the root Fe deficiency responses is (are) still unknown. Local Fe sensing is also a major task for roots, for adjusting the internal Fe requirements to external Fe availability: how such sensing is achieved and how it leads to metabolic adjustments in case of nutrient shortage, is mostly unknown. Two proteins belonging to the 2′-OG-dependent dioxygenases family accumulate several folds in Fe-deficient *Arabidopsis *roots. Such proteins require Fe(II) as enzymatic cofactor; one of their subgroups, the HIF-P4H (hypoxia-inducible factor-prolyl 4-hydroxylase), is an effective oxygen sensor in animal cells. We envisage here the possibility that some members of the 2′-OG dioxygenase family may be involved in the Fe deficiency response and in the metabolic adjustments to Fe deficiency or even in sensing Fe, in plant cells.

## INTRODUCTION

Iron is an essential micronutrient for plants although it is potentially toxic, when present in a free, non-complexed form. A recent review on that subject (Kobayashi and Nishizawa, 2012) details the knowledge accumulated on the regulation of plant Fe homeostasis, its intracellular trafficking and transport across cellular compartments and organs under various conditions of Fe supply, unveiling a complex net of molecular interactions. Beside the intensification of Fe-uptake strategies activated by plants under Fe-limiting conditions, root cells reprogram their metabolism to better cope with shortage of Fe (Vigani et al., 2012). Low Fe content triggers a high energy request to sustain the increased rate of Fe uptake from the soil, and at the same time it impairs the function of mitochondria and chloroplasts which provide energy to the cells. Thus, cells must increase the rate of alternative energy-providing pathways, such as glycolysis, Krebs cycle, or pentose phosphate pathway (López-Millán et al., 2000, 2012; Li et al., 2008; Vigani and Zocchi, 2009; Donnini et al., 2010; Rellán-Álvarez et al., 2010; Vigani, 2012a). To date, however, the sensors of plant “Fe status” triggering the signal transduction pathways, which eventually induce transcription factors such as the *Arabidopsis* FIT1, are still unknown and represent a challenging issue in plant science (Vigani et al., 2013). Efforts to fill up such gap of knowledge have been made by different research groups since years (Schmidt and Steinbach, 2000); recently, it has been demonstrated that localized Fe supply stimulates lateral root formation through the AUX1 auxin importer, which is proposed as a candidate for integrating the local Fe status in auxin signaling (Giehl et al., 2012).

## 2′-OG Fe(II)-DEPENDENT DIOXYGENASES AND PROLYL 4-HYDROXYLASES

It has been recently observed that some similarities might exist between the metabolic reprogramming occurring in Fe-deficient roots and that one occurring in tumor cells (Vigani, 2012b). In tumor cells, such reprogramming is known as “Warburg-effect” in which glucose is preferentially converted to lactate by enhancing glycolysis and fermentative reactions rather than completely oxidized by oxidative phosphorylation (OXOPHOS; Brahimi-Horn et al., 2007). Also in root cells a low Fe availability causes a decrease of OXOPHOS activity and induction of glycolysis and anaerobic reactions (Vigani, 2012b). The Warburg-effect in animal cells is mediated by hypoxia-inducible factor (HIF1), a heterodimeric complex whose α subunit is inducible by hypoxia. Under normoxic conditions, HIFα is post-translationally modified via the hydroxylation of proline residues by prolyl 4-hydroxylases (P4H); such modification leads to the proteasome-mediated degradation of HIFα. Under hypoxic conditions, however, such hydroxylation cannot occur because P4H enzymes belong to the 2-oxoglutarate Fe(II)-dependent dioxygenase family which have molecular oxygen and oxoglutarate as co-substrates; in other words, the lack of oxygen inhibits the P4H enzymatic activity, HIFα escapes degradation, it translocates to the nucleus where it can therefore form a dimer with HIFβ subunit; the complex then activates the cascade of hypoxia-responsive gene expression pathways (Myllyharju, 2003; Ken and Costa, 2006; Semenza, 2007).

Prolyl 4-hydroxylases are present in animal as well as in plant cells. In animal cells, P4H are classified into two categories: the collagen-type-P4H and the above cited HIF-P4H. The first class is localized within the lumen of the endoplasmic reticulum and it catalyzes the hydroxylation of proline residues within -X-Pro-Gly- sequences in collagen and in collagen-type proteins (Myllyharju, 2003), thus stabilizing their triple helical structure at body temperature (Myllyharju, 2003). These P4Hs are α_2_β_2_ tetramers and their catalytic site is located in the α subunit (Myllyharju, 2003; Tiainen et al., 2005). Three aa residues, His-412, Asp-414, and His-483, are the binding sites for Fe(II) in the human α(I) subunit (Myllyharju, 2003). The second class of P4H is localized in cytoplasm and it is responsible for hydroxylation of a proline residue in the HIFα subunit, under normoxic conditions, as described above. The *K*_m_ values of HIF-P4Hs for O_2_ are slightly above atmospheric concentration, making such proteins effective O_2_ sensors (Hirsilä et al., 2003). A novel role has also been uncovered for a human collagen-type-P4H, as regulator of Argonaute2 stability with consequent influence on RNA interference mechanisms (Qi et al., 2008).

Several genes similar to P4H are present in plants; for instance, 13 P4H have been identified in *Arabidopsis* and named AtP4H1–AtP4H13 (Vlad et al., 2007a,b); with the exception of AtP4H11 and AtP4H12, the three binding residues for Fe(II) (two His and one Asp) as well as the Lys residues binding the 2-oxoglutarate, are all conserved in such P4Hs (Vlad et al., 2007a). The different isoforms are more expressed in roots than in shoots and they show different pattern of expression in response to various stresses (hypoxia, anoxia, and mechanical wounding ; Vlad et al., 2007a,b).

Cloning and biochemical characterization of two of them, i.e., AtP4H1, encoded by At2g43080 gene (Hieta and Myllyharju, 2002) and At4PH2, encoded by At3g06300 gene (Tiainen et al., 2005) show that substrate specificity varies: recombinant AtP4H1 effectively hydroxylates poly(L-proline) and other synthetic peptides with *K*_m_ values lower than those for AtP4H2, thus suggesting different physiological roles between the two. Recombinant AtP4H1 can also effectively hydroxylate human HIFα-like peptides and collagen-like peptides, whereas recombinant AtP4H2 cannot (Hieta and Myllyharju, 2002; Tiainen et al., 2005). Their *K*_m_ for Fe(II) are 16 and 5 μM, respectively (Hieta and Myllyharju, 2002; Tiainen et al., 2005).

Two proteins belonging to the 2′-OG dioxygenase family, encoded by At3g12900 and At3g13610 genes, accumulate several folds in Fe-deficient roots, when compared to Fe-sufficient ones (Lan et al., 2011). The protein encoded by At3g13610 gene, named F6′H1, is involved in the synthesis of coumarins via the phenylpropanoid pathway, as it catalyzes the ortho-hydroxylation of feruloyl CoA, which is the precursor of scopoletin (Kai et al., 2008). Scopoletin and its β-glucoside scopolin accumulate in *Arabidopsis *roots and, at lower levels, also in shoots (Kai et al., 2006).

A severe reduction of scopoletin levels can be observed in the KO mutants for the At3g13610 gene (Kai et al., 2008). One of the responses to Fe deficiency, is the induction of the phenylpropanoid pathway (Lan et al., 2011). Phenolics can facilitate the reutilization of root apoplastic Fe (Jin et al., 2007a,b) and a phenolic efflux transporter PEZ1 located in the stele has been identified in rice (Ishimaru et al., 2011). The secretion of phenolic compounds can, moreover, selectively modify the soil microbial population in the surroundings of the roots, which in turn can favor acquisition of Fe by production of siderophores as well as auxin-like compounds (Jin et al., 2006, 2008, 2010).

Plant 2′-OG dioxygenases are also involved in synthesis of phytosiderophores such as Ids3 from barley, which is induced by Fe deficiency and it catalyzes the hydroxylation step from 2′-deoxymugeinic acid (DMA) to mugeinic acid (MA; Kobayashi et al., 2001).

## 2′-OG Fe(II)-DEPENDENT DIOXYGENASES, Fe DEFICIENCY RESPONSE AND METABOLIC REPROGRAMMING: IS THERE A COMMON LINK?

**FIGURE 1:**
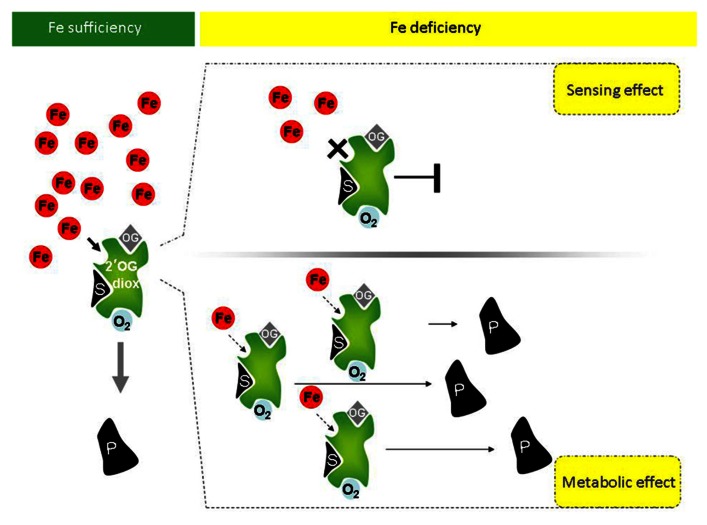
**Potential involvement of plant 2′-OG-dioxygenases in Fe sensing, in the Fe deficiency responses and in the metabolic reprogramming occurring under Fe deficiency**. Under Fe sufficiency, in presence of the co-factors O_2_ and oxoglutarate (OG), 2′-OG-dioxygenases (in green) can catalyze the dioxygenase reaction leading to production of product P from substrate S. Under Fe deficiency, however, at least two scenarios can occur, depending on the *K*_m_ of a given 2′-OG-dioxygenase for Fe. If the *K*_m_ is close to the physiological concentration of the labile iron pool (LIP; free redox-active Fe ions), upon reduction of Fe availability, the enzymatic activity of the 2′-OG-dioxygenase is drastically reduced or fully inhibited and the reduction or complete lack of enzymatic product can be itself a signal of “Fe deficiency” triggering the Fe deficiency response cascades (upper panel, right). If instead *K*_m_ is far below the physiological LIP, the enzyme might still be active (lower panel, right). Not only, if transcriptional/translational up-regulation of such 2′-OG-dioxygenase takes place under Fe deficiency, then an increased total enzymatic activity can lead to higher production of product P (lower panel, right). Product P, in turn, could be involved in the Fe response/metabolic adjustments occurring under Fe deficiency.

Given the above premises, it is possible that a link among P4H activity, and more generally among 2′-OG Fe(II)-dependent dioxygenase activities, the Fe deficiency responses and the metabolic reprogramming occurring during Fe deficiency exists in higher plants. If such a link exists for a given 2′-OG Fe(II)-dependent dioxygenase, at least two possible scenarios could be predicted for such enzyme (**Figure 1**):

(a) If, for a given sub-cellular localization, the *K*_m_ of such enzyme for Fe is close to the physiological concentration of the LIP (labile iron pool, consisting of free redox-active Fe ions), then the enzyme activity is strongly affected by Fe fluctuations, similarly to the above described HIF-P4H, which is an effective sensor for O_2_ (Hirsilä et al., 2003). Upon reduction of Fe availability below the physiological LIP, its enzymatic activity should be indeed drastically reduced or fully inhibited; reduction or complete lack of enzymatic product might, in turn, triggers the “Fe deficiency” signaling. The enzyme might therefore act as true Fe sensor (**Figure 1**, upper panel, right).

Although the Fe-dependent transcriptional regulation of such an Fe sensor enzyme might be not expected, it cannot be excluded *a priori*: for example, chitin recognition is dependent not only on the presence of specific receptors, but also on the expression of extracellular chitinases, which are essential for the production of smaller chito-oligosaccharides from chitin hydrolysis, in animal (Gorzelanny et al., 2010; Vega and Kalkum, 2012) as well as in plant systems (Shibuya and Minami, 2001; Wan et al., 2008). These smaller, diffusible molecules induce, in turn, the expression of several defense protein, among which also chitinase activities. Chitinase is thus both an example of a crucial enzyme for the signal production but also an integral part of the response.

(b) If, for a given sub-cellular localization, the *K*_m_ for Fe of such an enzyme is instead far below the physiological LIP, the enzyme might still be active under Fe deficiency. Additionally, if transcriptional/translational up-regulation occurs under Fe deficiency, accumulation of protein and increased total enzymatic activity might be observed. The enzyme might be involved in the Fe response/metabolic adjustment occurring under Fe deficiency, without being itself a Fe sensor (**Figure 1**, lower panel, right).

This second scenario is supported by the evidence that the *Arabidopsis* 2′-OG-dioxygenase F6′H1 (described in previous paragraph) which accumulates in Fe-deficient roots (Lan et al., 2011) is indeed possibly involved in the Fe response/metabolic adjustment occurring under Fe deficiency: *Arabidopsis* mutants KO for the At3g13610 gene (coding for F6′H1) have indeed altered root phenotype under Fe deficiency (I. Murgia, unpublished observations).

Such a link among 2′-OG Fe(II)-dependent dioxygenase activity, the Fe deficiency responses and the metabolic reprogramming occurring during Fe deficiency can be explored first by analyzing the transcriptional co-regulation of 2′-OG-dependent dioxygenase genes with genes involved in the Fe deficiency response or in the metabolic reprogramming. The bioinformatic approach of our choice was already described (Beekwilder et al., 2008; Menges et al., 2008; Berri et al., 2009; Murgia et al., 2011) and successfully applied in *Arabidopsis* and rice. Such analysis identifies genes which are co-regulated in large microarray datasets; in this case, it provides candidate genes potentially involved in Fe metabolism, among the 2′-OG-dioxygenase family members. Although transcript levels do not equal protein levels (or activities), there is nevertheless evidence for correlation between the two in many organisms (Vogel and Marcotte, 2012). This approach is not only simple on a genomic scale, but it has proved useful to identify candidate genes in the past, which were then validated by experimental approaches (e.g., Beekwilder et al., 2008; Murgia et al., 2011; Møldrup et al., 2012).

*Arabidopsis* possesses almost one hundred annotated 2′-OG dioxygenase genes which make such analysis not immediate; we therefore restricted the analysis to the AtP4H subclass (with the exclusion of AtP4H8, AtP4H12, and AtP4H13 because the corresponding genes were not available in the Affymetrix microarray data set most commonly used). As pivot bioinformatic analysis, we analyzed the correlation of such AtP4H subclass with two gene groups. The first group consisted of a list of 25 Fe-homeostasis/trafficking/transport related genes, described in recent reviews on this subject (Conte and Walker, 2011; Kobayashi and Nishizawa, 2012). The second group consisted of an equal number of genes coding for enzymes possibly involved in the metabolic adjustments under Fe deficiency, such as those catalyzing the synthesis of pyruvate (Pyr). It is indeed known that several glycolitic genes are overexpressed in roots of Fe-deficient plants (Thimm et al., 2001): different isoforms of hexokinase (HXK), phosphoglyceratekinase, enolase (ENO), phosphoglycerate mutase (iPGAM), were therefore considered. Also, genes coding for enzymes involved in the consumption of Pyr by non-OXOPHOS reactions and whose expression is affected by Fe deficiency, such as alcohol dehydrogenase (ADH), lactate dehydrogenase (LDH), and malate dehydrogenase, were also considered (Thimm et al., 2001). Last, the genes coding for the four isoforms of *Arabidopsis *phospho*enol*pyruvate carboxylase (PEPC; PPC1, 2, 3, 4; Sanchez et al., 2006) were also included in the second group, since PEPC is strongly induced in several dicotyledonous plants under Fe deficiency (Vigani, 2012a) and PEPC is supposed to play a central role in the metabolic reprogramming occurring in Fe-deficient root cells (Zocchi, 2006).

As positive controls, the two 2′-OG-dioxygenases encoded by At3g12900 and At3g13610 and accumulating in Fe-deficient roots (Lan et al., 2011) whereas, as negative control, the ferritin gene whose expression is known to be repressed under Fe deficiency (Murgia et al., 2002), were included. The full list of genes for which the correlation analysis has been performed, is reported in **Table 1**.

**Table 1:** List of genes for which the correlation analysis with 2′-OG-dependent dioxygenases has been performed.

Fe homeostasis genes		Metabolic genes	
IRT1	At4g19690	HXK1	At4g29130
IRT2	At4g19680	HXK2	At2g19860
AHA2	At4g30190	HXK4	At3g20040
NAS1	At5g04950	HKL1	At1g50460
NAS2	At5g56080	HXL3	At4g37840
NAS3	At1g09240	PPC1	At1g53310
NAS4	At1g56430	PPC2	At2g42600
CYP82C4	At4g31940	PPC3	At3g14940
IREG2	At5g03570	PPC4	At1g68750
MTP3	At3g58810	PGK	At1g79550
Popeye	At3g47640	PGK1	At3g12780
Brutus	At3g18290	LDH	At4g17260
NRAMP3	At2g23150	ENO1	At1g74030
NRAMP4	At5g67330	ENOC	At2g29560
FRO3	At1g23020	ENO2	At2g36530
FRO7	At5g49740	iPGAM	At1g09780
FRD3	At3g08040	PGM	At1g78050
ILR3	At5g54680	PDC2	At5g54960
YSL1	At4g24120	PDC3	At5g01330
ZIF1	At5g13740	G6PD4	At1g09420
VIT1	At2g01770	MMDH2	At3g15020
Fer1	At5g01600	mal dehydr family	At3g53910
Fer2	At3g11050	mal dehydr family	At4g17260
Fer3	At3g56090	mal dehydr family	At5g58330
Fer4	At2g40300	ADH1	At1g77120
		ADH transcrip factor	At2g44730
		ADH transcrip factor	At3g24490

*Genes in the left column are known to be involved in the Fe deficiency response, in regulation of Fe homeostasis or Fe trafficking. Genes in the right column are involved in glycolysis or in the consumption of pyruvate by non-OXOPHOS reactions; genes coding for the four isoforms of Arabidopsis phosphoenolpyruvate carboxylase (PEPC; PPC1, 2, 3, 4) have been also included. IRT, iron-regulated transporter; AHA, Arabidopsis H^+^-ATPase; NAS, nicotianamine synthase; CYP82C4, cytochrome P450 82C4; IREG, ferroportin/iron-regulated; MTP3, metal tolerance protein; NRAMP, natural resistance-associated macrophage protein; FRO, ferric-chelate oxidase reductase; FRD, ferric reductase defective; ILR, IAA-leucine resistant; YSL, yellow stripe-like; ZIF, zinc induced facilitator; VIT, vacuolar iron transporter; Fer, ferritin; HXK1, hexokinase; HXL, hexokinase-like; PPC, phosphoenolpyruvate carboxylase; PGK, phosphoglyceratekinase; LDH, lactate dehydrogenase; ENO, enolase; ENOC, cytosolic enolase; iPGAM, phosphoglycerate mutase; PGM, phosphoglycerate/bisphosphoglycerate mutase; PDC, pyruvate decarboxylase; G6PD4, glucose-6-phosphate dehydrogenase; MMDH, mitochondrial malate dehydrogenase; mal dehydr family, malate dehydrogenase family; ADH, alcohol dehydrogenase.*

The resulting Pearson’s correlation coefficients, calculated by using either linear or logarithmic expression values (Menges et al., 2008; Murgia et al., 2011) are reported in **Table 2**, if above a defined threshold (≥0.60 or ≤-0.60); genes for which none of the Pearson’s coefficient fulfilled this condition, were not included in **Table 2** (AtP4H3, AtP4H9, AtP4H10 and AtP4H11).

**Table 2:** Correlation analysis of Arabidopsis thaliana 2′-OG dioxygenase genes with genes involved in Fe deficiency response or with genes possibly involved in metabolic reprogramming during Fe deficiency.

		2′-OG-dioxyg		2′-OG-dioxyg		P4H-1		P4H-2		P4H-4		P4H-5		P4H-6	
	AGI code	At3g12900		At3g13610		At2g43080		At3g06300		At5g18900		At2g17720		At3g28490	
		lin	log	lin	log	lin	log	lin	log	lin	log	lin	log	lin	log
IRT1	At4g19690	**0.69**	0.23	**0.74**	**0.69**	0.21	0.21	0.54	0.55	0.10	0.11	0.36	0.53	-0.02	0.05
AHA2	At4g30190	0.22	0.09	**0.67**	**0.69**	0.35	0.31	**0.76**	**0.71**	0.18	0.22	**0.76**	**0.79**	-0.07	-0.07
CYP82C4	At4g31940	0.54	0.47	**0.61**	0.56	0.30	0.33	0.40	0.43	0.16	0.22	0.27	0.37	-0.04	-0.13
IREG2	At5g03570	**0.73**	0.42	**0.79**	0.59	0.26	0.22	**0.64**	0.55	0.23	0.31	0.41	0.51	-0.02	-0.08
MTP3	At3g58810	**0.77**	0.27	**0.83**	**0.70**	0.30	0.37	**0.62**	**0.66**	0.16	0.23	0.40	0.58	-0.04	-0.10
HXK4	At3g20040	0.25	0.22	0.32	0.25	0.24	0.27	0.29	0.37	0.26	0.02	0.17	0.26	**0.72**	0.06
HXL3	At4g37840	0.01	0.05	-0.06	-0.03	0.13	0.05	-0.08	-0.06	0.32	0.11	-0.05	-0.06	**0.66**	0.35
PPC1	At1g53310	0.03	-0.06	0.41	0.55	0.19	0.22	**0.60**	**0.63**	0.03	0.10	**0.73**	**0.70**	-0.10	-0.06
PPC3	At3g14940	0.23	0.21	**0.78**	**0.73**	0.35	0.27	**0.69**	0.58	0.19	0.23	0.58	0.58	0.02	0.03
PPC4	At1g68750	0.01	0.08	-0.04	0.10	0.13	0.05	-0.05	0.08	0.35	0.08	0.00	0.13	**0.78**	0.21
PGK1	At3g12780	-0.13	-0.11	-0.50	-0.41	-**0.67**	-**0.69**	-0.49	-0.46	-**0.66**	-**0.74**	-0.33	-0.30	-0.09	-0.03
ENO1	At1g74030	0.14	0.03	**0.72**	0.59	0.37	0.31	**0.67**	0.59	0.12	0.06	0.54	0.49	-0.05	-0.01
iPGAM	At1g09780	0.07	0.03	0.47	0.51	0.04	0.10	0.54	0.56	-0.21	-0.32	**0.61**	**0.62**	-0.07	-0.03
Mal. d. fam.	At5g58330	-0.19	-0.23	-0.56	-0.49	-**0.70**	-**0.67**	-**0.61**	-**0.61**	-0.59	-**0.60**	-0.47	-0.47	-0.08	0.02

*For each gene pair, the Pearson’s correlation coefficient, from logarithmic or linear analysis, is reported. Coefficients with values ≥0.60 or ≤-0.60 are highlighted in gray.*

In accordance with results obtained by iTRAQ (isobaric peptide tags for relative and absolute quantitation) analysis of Fe-deficient roots (Lan et al., 2011), both At3g12900 and At3g136100 show positive correlation with genes actively involved in the Fe deficiency response, such as iron-regulated transporter 1 (IRT1; Vert et al., 2002), ferric-chelate oxidase reductase (FRO2; Connolly et al., 2003) CYP82C4 (Murgia et al., 2011), ferroportin/iron-regulated (IREG2; Morrissey et al., 2009) metal tolerance protein (MTP3; Arrivault et al., 2006)(**Table 2**); viceversa, they show no significant correlation with the ferritin genes since their correlation values fall within the [-0.3 + 0.02] range (data not shown).

According to such results, the AtP4H genes could be divided into three classes:

Class 1: positive or negative correlation with metabolic genes only (At3g28490, At2g43080, and At5g18900).
Class 2: positive correlation with Fe-related genes and positive or negative correlation with metabolic genes (At2g17720 and At3g06300, beside the positive control At3g13610).
Class 3: no significant correlation (positive or negative) with any of the genes tested (At1g20270, At4g33910, At5g66060, At4g35820).

Genes in class 1 might be not involved in the plant response to improve Fe uptake and trafficking in order to alleviate Fe deficiency symptoms.

Genes in class 2 might be the ones linking the stimulation of Fe deficiency response with the metabolic adaptations triggered by Fe deficiency (**Figure 1**) whereas genes in class 3 might contain the candidate Fe sensor(s) (**Figure 1**).

Regarding the genes in class 2, it is interesting to notice that beside with the Fe-related genes, the positive control At3g13610 is positively correlated with PPC3 and ENO1, the At3g06300 gene coding for P4H2 (Tiainen et al., 2005) is positively correlated with PPC1, PPC3, ENO1, and also negatively correlated with a malate dehydrogenase family member whereas the At2g17720 gene coding for P4H5 is positively correlated with PPC1 and iPGAM (Zhao and Assmann, 2011).

Interestingly, PPC1 and PPC3 are mainly expressed in root tissues and their expression is affected by abiotic stress when compared with PPC2, which is considered to cover an housekeeping role (Sanchez et al., 2006), whereas ENO1 encodes the plastid-localized isoform of phospho*enol*pyruvate (PEP)-ENO (Prabhakar et al., 2009); PEP is further metabolized to Pyr by pyruvate kinase (PK). PEP and Pyr represent essential precursors for anaerobic reaction. PEP is fed into the schikimate pathway, which is localized within the plastid stroma (Herrmann and Weaver, 1999) and which is essential for a large variety of secondary products. Pyr can also act as precursor for several plastid-localized pathways, among which the mevalonate-independent way of isoprenoid biosynthesis (Lichtenthaler, 1999). Plastid-MEP (2-*C*-methyl-D-erythritol 4-phosphate) pathways might be responsible for the synthesis of a signal molecule putatively involved in the regulation of Fe homeostasis (Vigani et al., 2013).

Such analysis is preliminary and needs to be extended to all 2′-OG-dioxygenase gene family members. Genes candidate as Fe sensors can be further analyzed experimentally, insofar that loss-of function mutants lacking the “Fe sensing” function should display a Fe deficiency response, even in Fe-sufficient conditions (see **Figure 1**, upper panel, right).

## FUTURE DIRECTIONS

Elucidation of nutrient sensing and signaling is a major issue in plant physiology and crop production, with potential impact in the design of new biofortification strategies for improving yields as well as the nutritional value of crops of interest (Murgia et al., 2012; Schachtman, 2012). In *Arabidopsis*, a major sensor of nitrate is the nitrate transporter NRT1.1, which is the first representative of plant “transceptors,” thus indicating their dual nutrient transport/signaling function (Gojon et al., 2011). Transceptors, whose feature is that transport and sensing activity can be uncoupled, have been described in animals and yeasts (Thevelein and Voordeckers, 2009; Kriel et al., 2011) and more active transceptors have been postulated also in plants (Gojon et al., 2011).Three major global challenges faced by agriculture are food and energy production as well as environmental compatibility (Ehrhardt and Frommer, 2012). Advancements in the area of nutrient sensing and signaling can positively contribute solutions to all these three challenges and the extensive analysis of the complete 2′-OG-dioxygenase gene family, based on pilot analysis described in the present perspective, could be a novel way to pursue these advancements.

## Conflict of Interest Statement

The authors declare that the research was conducted in the absence of any commercial or financial relationships that could be construed as a potential conflict of interest.
